# Resistance profile and mechanism of severe acute respiratory syndrome coronavirus-2 variants to LCB1 inhibitor targeting the spike receptor-binding motif

**DOI:** 10.3389/fmicb.2022.1022006

**Published:** 2022-10-11

**Authors:** Tong Wu, Yuanmei Zhu, Nian Liu, Yue Hu, Huihui Chong, Yuxian He

**Affiliations:** NHC Key Laboratory of Systems Biology of Pathogens, Center for AIDS Research, Institute of Pathogen Biology, Chinese Academy of Medical Sciences and Peking Union Medical College, Beijing, China

**Keywords:** SARS-CoV-2, variants of concern, entry inhibitor, LCB1, drug resistance

## Abstract

LCB1 is a 56-mer miniprotein computationally designed to target the spike (S) receptor-binding motif of SARS-CoV-2 with potent *in vitro* and *in vivo* inhibitory activities (Cao et al., 2020; Case et al., 2021). However, the rapid emergence and epidemic of viral variants have greatly impacted the effectiveness of S protein-targeting vaccines and antivirals. In this study, we chemically synthesized a peptide-based LCB1 inhibitor and characterized the resistance profile and underlying mechanism of SARS-CoV-2 variants. Among five variants of concern (VOCs), we found that pseudoviruses of Beta, Gamma, and Omicron were highly resistant to the LCB1 inhibition, whereas the pseudoviruses of Alpha and Delta as well as the variant of interest (VOI) Lambda only caused mild resistance. By generating a group of mutant viruses carrying single or combination mutations, we verified that K417N and N501Y substitutions in RBD critically determined the high resistance phenotype of VOCs. Furthermore, a large panel of 85 pseudoviruses with naturally occurring RBD point-mutations were generated and applied to LCB1, which identified that E406Q, K417N, and L455F conferred high-levels of resistance, when Y505W caused a ∼6-fold resistance fold-change. We also showed that the resistance mutations could greatly weaken the binding affinity of LCB1 to RBD and thus attenuated its blocking capacity on the interaction between RBD and the cell receptor ACE2. In conclusion, our data have provided crucial information for understanding the mechanism of SARS-CoV-2 resistance to LCB1 and will guide the design strategy of novel LCB1-based antivirals against divergent VOCs and evolutionary mutants.

## Introduction

Severe acute respiratory syndrome coronavirus-2 (SARS-CoV-2) caused the global pandemic of coronavirus disease 2019 (COVID-19), which has recently resulted in more than 551 million confirmed cases with about 6.4 million deaths.^1^ During the spread of the virus, many variants of concern (VOCs) emerged with significantly changed infectivity and pathogenicity, leading to new waves of infection that have posed daunting challenges (Callaway, 2021; Garcia-Beltran et al., 2021; He et al., 2021; Hoffmann et al., 2021; Kuzmina et al., 2021). The previous four VOCs are Alpha (B.1.1.7), Beta (B.1.351), Gamma (P.1), and Delta (B.1.617.2); the fifth one, Omicron (B.1.1.529), was first reported from Southern Africa in late November 2021. Genome-sequencing data indicate that Omicron variant evolves with the largest number of mutations, including 32 mutations located within spike (S) protein that cover the key mutations in the receptor binding domain (RBD) or motif (RBM), such as K417N, E484A, and N501Y (Callaway, 2021; He et al., 2021; Tao et al., 2021; Yamasoba et al., 2022). Significantly, Omicron is highly transmissible and can spread several times faster than any previous variants; thus, it has quickly outcompeted Delta variant to dominate the epidemic, caused a large number of breakthrough infection or re-infection, and seriously impaired the clinical efficacies of preventive vaccines and therapeutic antibodies (He et al., 2021; Guo et al., 2022; Markov et al., 2022).

Since the outbreak of COVID-19, many efforts have been devoted to the development of antivirals that block different steps of SARS-CoV-2 life-cycle, including viral entry, replication, assembly, budding, and releasing (Yang and Rao, 2021; Yan et al., 2022). Notably, virus entry inhibitors that can inhibit the interaction between S protein and the human cellular receptor angiotensin-converting enzyme 2 (ACE2) are considered a promising strategy (Plavec et al., 2021; Sabbah et al., 2021; Xiang et al., 2021). By applying computational *de novo* design approaches, Cao and coworkers developed a group of miniproteins, which bound the spike RBD with affinities ranging from 100 pM to 10 nM and inhibited SARS-CoV-2 infection with 50% inhibitory concentration (IC_50_) values between 24 pM and 35 nM (Cao et al., 2020). LCB1, a lead miniprotein designed with 56 amino acids, showed the potent *in vitro* antiviral activity, and its modified versions efficiently blocked SARS-CoV-2 infection in human ACE2 (hACE2)-expressing transgenic mice when administrated as both pre-exposure prophylaxis (PrEP) and post-exposure therapy, providing an ideal candidate for drug development (Case et al., 2021). Considering the COVID-19 epidemic caused by divergent VOCs and still ongoing evolutionary mutations, it is fundamentally important to characterize LCB1 for its drug resistance and underlying mechanism. In this report, we describe our data to specifically address this question, which can guide the design strategy of novel LCB1-based antivirals against divergent VOCs and evolutionary mutants.

## Materials and methods

### Peptide, plasmids, and cell lines

A 56-mer LCB1 peptide was synthesized on rink amide 4-methylbenzhydrylamine (MBHA) resin using a standard solid-phase 9-flurorenylmethoxycarbonyl (FMOC) protocol as described previously (Yu et al., 2021a). Plasmids encoding the mutant S proteins of SARS-CoV-2 (Alpha, Beta, Gamma, Delta, Lambda, and Omicron) were a kind gift from Linqi Zhang at the Tsinghua University (Beijing, China). HEK293T and Huh-7 cells were purchased from the American type culture collection (ATCC) (Rockville, MD, USA); 293T/ACE2 cells stably expressing human ACE2 were generated and preserved in our laboratory. Cells were cultured in complete growth medium consisting of Dulbecco’s minimal essential medium (DMEM) supplemented with 10% fetal bovine serum (FBS), 100 U/ml of penicillin-streptomycin, 2 mM L-glutamine, and 1mM sodium pyruvate under 37^*o*^C and 5% CO2.

### Circular dichroism spectroscopy

Circular dichroism (CD) spectroscopy was applied to determine the secondary structure and thermostability of LCB1 peptide as described previously (Zhu et al., 2020). Briefly, LCB1 was dissolved in phosphate-buffered saline (PBS; pH 7.2) with a final concentration of 20 μM and incubated at 37^*o*^C for 30 min. CD spectra were obtained on Jasco spectropolarimeter (model J-815) a using a 1 nm bandwidth with a 1 nm step resolution from 195 to 270 nm at room temperature. The spectra were corrected by subtracting a solvent blank, and the α-helical content was calculated from the CD signal by dividing the mean residue ellipticity [θ] at 222 nm by with a value of −33,000 deg cm^2^ dmol^–1^, corresponding to a 100% helix. Thermal denaturation was done by monitoring the ellipticity change at 222 nm from 20 to 98^*o*^C at a rate of 2^*o*^C/min, and the melting temperature (*T*_*m*_) was defined as the midpoint of the thermal unfolding transition.

### Site-directed mutagenesis

Spike mutants were generated by site-directed mutagenesis as described previously (Yu et al., 2021a). In brief, the forward and reverse primers with 16∼28 nucleotides were designed with specific mutations and occupied the same starting and ending positions on the opposite strands of a codon-optimized S gene cloned in a pcDNA3.1 vector. DNA synthesis was conducted by PCR in a 50-μl reaction volume using 100 ng of denatured plasmid template, 50 pM upper and lower primers, and 5 U of the high-fidelity polymerase PrimeStar (TaKaRa, Dalian, China). PCR amplification was done for one cycle of denaturation at 98°C for 5 min, followed by 25 cycles of 98°C for 10 s and 68°C for 9 min, with a final extension at 72°C for 10 min. The amplicons were treated with restriction enzyme *Dpn*I for 3 h at 37°C, and *Dpn*I-resistant molecules were recovered by transforming *E. coli* stable3 competent cells with antibiotic resistance. The required mutations were confirmed by DNA sequencing of a single clone.

### Single-cycle infection assay

Infectivity of various SARS-CoV-2 pseudoviruses on 293T/ACE2 or Huh-7 cells was measured by a single-cycle infection assay as described previously (Yu et al., 2021a). Briefly, pseudoviruses were packaged by cotransfecting HEK293T cells with a wild-type (WT) or mutant S protein-expressing plasmid and pNL4-3.luc.RE plasmid that encodes an Env-defective HIV-1_*NL*4–3_ genome with luciferase as a reporter. Cell supernatants containing virions were collected after transfection 48 h and stored at −80^*o*^C. To determine the inhibitory activity of LCB1 inhibitor, a serially three-fold diluted peptide was incubated with an equal volume of pseudoviruses at 37^*o*^C for 1 h, and the peptide-virus mixture was then added to 293T/ACE2 or Huh-7 target cells at a density of 10^4^ cells/100 μl per well in a 96-well culture plate. After incubation at 37^*o*^C for 48 h, cells were harvested, lysed in reporter lysis buffer, and measured for luciferase activity using luciferase assay reagents and a luminescence counter (Promega, Madison, WI, USA).

### Biolayer interferometry

Biolayer interferometry (BLI) was used to measure the binding and blocking activities of LCB1 peptide. In brief, a recombinant His-tagged RBD protein (Sino Biological, Beijing, China) was loaded on a NTA biosensor (ForteBio, San Francisco, CA, USA) at a concentration of 10 μg/ml for 120 s in phosphate buffer saline (PBS), and LCB1 peptide was gradient-diluted in PBST buffer (PBS plus 0.2% Tween 20). The binding kinetics was guided by associating in analyte substrates for 120s and disassociating in PBST alone for 300s.

### Flow cytometry assay

The blocking activity of LCB1 peptide on the interaction of ACE2 and RBD proteins was also determined by flow cytometry. Briefly, an RBD protein at 2 μg/ml was mixed with serially diluted LCB1 and incubated at 4°C for 1 h. The mixture was then added to 5 × 10^5^ of 293T/ACE2 cells and incubated at 4°C for 1 h. After being washed twice with PBS, the cells were incubated with 1:500 diluted Alexa Fluor 488-labeled rabbit anti-His tag antibody (Cell Signaling Technology, Danvers, MA, USA) at 4°C for 1 h. After two washes with PBS, cells were resuspended by FACS buffer and analyzed with a FACS CantoII instrument (Becton-Dickinson, Mountain View, CA, USA).

### Statistical analysis

The percent inhibition of virus infection and 50% inhibitory concentration (IC_50_) of LCB1 inhibitor were calculated using GraphPad Prism 6 software (GraphPad Software Inc., San Diego, CA, USA). Statistical comparison of divergent pseudovirus infections were conducted by one-way ANOVA with Dunnett’s multiple comparisons test (^∗^*P* < 0.05; ^∗∗^*P* < 0.01; ^∗∗∗^*P* < 0.001; ^∗∗∗∗^*P* < 0.0001; ns, not significant).

## Results

### Synthesis and characterization of LCB1 peptide

**FIGURE 1:**
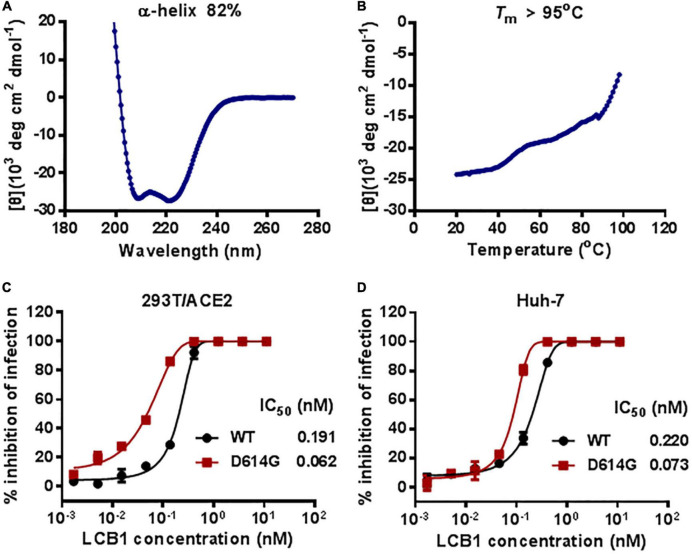
Structural and functional characterization of LCB1 peptide. The α-helicity **(A)** and thermostability **(B)** of LCB1 peptide at a concentration of 20 μM were determined by Circular dichroism (CD) spectroscopy. The inhibitory activities of LCB1 against infections of wild-type (WT) SARS-CoV-2 and its D614G mutant on 293T/ACE2 cells **(C)** and Huh-7cells **(D)** were measured by a single-cycle infection assay. Samples were tested in triplicate and repeated, and data are presented as means.

The previously reported LCB1 miniprotein was recombinantly expressed and purified from *E. coli*. Herein, we chemically synthesized a 56-mer LCB1 peptide using a standard solid-phase FMOC protocol. The peptide was acetylated at the N terminus and amidated at the C terminus, purified to a 95.15% homogeneity by reverse-phase high-performance liquid chromatography (HPLC) and characterized for amino acids with mass spectrometry (Supplementary Figure 1). The concentration of LCB1 peptide was determined by UV absorbance and a theoretically calculated molar extinction coefficient based on the tryptophan and tyrosine residues. We first characterized the structural properties of LCB1 by CD spectroscopy. As shown in Figures 1A,B, CD spectra of LCB1 displayed typical double minima at 208 and 222 nm, which indicated an α-helical content of ∼82%; however, its thermal unfolding transition could not be precisely determined due to a greater than 95^*o*^C melting temperature (*T*_*m*_). Next, the antiviral activity of LCB1 was measured by a pseudovirus-based single-cycle infection assay. To this end, pseudoviruses carrying wild-type (WT) S protein of the original SARS-CoV-2 Wuhan-Hu-1 strain or a single D614G mutation (B1 strain) were packaged and characterized. As shown in Figures 1C,D, LCB1 potently inhibited infections of the WT and D614G pseudoviruses with mean IC_50_ values of 0.191 and of 0.062 nM, respectively, on 293T/ACE2 cells and of 0.22 and 0.073 nM, respectively, on Huh-7 cells. In comparison, LCB1 was ∼3-fold more active in inhibiting the D614G mutant relative to its inhibition on WT strain. Taken together, these results validated the structural integrity and functionality of LCB1 as a peptide-based inhibitor.

### Resistance profiles of diverse variants of concerns and variant of interest to LCB1 inhibitor

**FIGURE 2:**
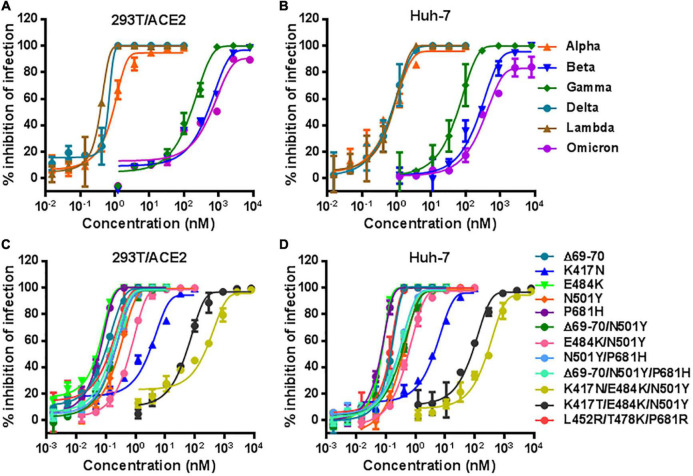
Inhibitory activity of LCB1 against divergent SARS-CoV-2 variants. The activities of LCB1 peptide in inhibiting VOCs and VOI **(A,B)** as well as the panel of mutant pseudoviruses carrying single or combined mutations **(C,D)** were measured by the single-cycle infection assay. Samples were tested in triplicate, the experiments were repeated three times, and data are expressed as the means with standard deviations (SD) and presented in Table 1.

We recently reported the functionalities of S proteins derived from divergent SARS-CoV-2 variants to mediated cell-cell fusion and infectivity, as well as their susceptibility to the inhibition of fusion-inhibitory lipopeptides. Herein, we sought to characterize LCB1 peptide for its inhibitory activity on five VOCs and one VOI (Lambda, C.37). The corresponding pseudoviruses were therefore generated and used in the single-cycle infection assay. As shown in Figures 2A,B and Table 1, LCB1 infected Alpha, Beta, Gamma, Delta, Lambda, and Omicron variants with mean IC_50_ values of 0.899, 901.8, 204.367, 0.569, 0.301, and 956.5 nM, respectively, on 293T/ACE2 cells and of 0.69, 447.083, 70.405, 0.958, 0.47, and 761.217 nM, respectively, on Huh-7 cells. Because all the VOCs were evolved with a D614G background, here we used D614G virus as a reference strain for calculating the fold-changes of resistance. As shown, Alpha, Beta, Gamma, Delta, Lambda, and Omicron displayed about 15-, 14, 545-, 3, 296-, 9-, 5-, and 15,427-fold resistance on 293T/ACE2 cells and about 9-, 6, 124-, 964-, 13-, 6-, and 10,428-fold resistance on Huh-7 cells, respectively. Therefore, the Beta, Gamma, and Omicron variants had very high levels of resistance to the LCB1 inhibition, whereas the Alpha, Delta, and Lambda variants exhibited mild resistance.

**TABLE 1:** Resistance profile of SARS-CoV-2 VOCs and related mutants to LCB1 inhibitor^a^.

Pseudovirus	PV infection on 293T/ACE2		PV infection on Huh-7	
	IC_50_ ± SD (nM)	Fold change	IC_50_ ± SD (nM)	Fold change
D614G	0.062 ± 0.001	1	0.073 ± 0.008	1
Alpha	0.899 ± 0.139	14.500	0.69 ± 0.075	9.452
Beta	901.8 ± 68.826	14545.161	447.083 ± 1.061	6124.425
Gamma	204.367 ± 0.189	3296.242	70.405 ± 17.067	964.452
Delta	0.569 ± 0.133	9.177	0.958 ± 0.319	13.123
Lamda	0.301 ± 0.078	4.855	0.47 ± 0.029	6.438
Omicron	956.5 ± 43.982	15427.419	761.217 ± 69.367	10427.630
Δ69-70	0.101 ± 0.011	1.629	0.137 ± 0.005	1.877
K417N	2.774 ± 0.191	44.742	4.898 ± 0.357	67.096
E484K	0.059 ± 0.001	0.952	0.07 ± 0.013	0.959
N501Y	0.228 ± 0.022	3.677	0.282 ± 0.037	3.863
P681H	0.071 ± 0.002	1.145	0.069 ± 0.011	0.945
Δ69-70/N501Y	0.209 ± 0.004	3.371	0.392 ± 0.032	5.370
E484K/N501Y	0.579 ± 0.069	9.339	0.525 ± 0.15	7.192
N501Y/P681H	0.199 ± 0.014	3.210	0.31 ± 0.048	4.247
Δ69-70/N501Y/P681H	0.148 ± 0.03	2.387	0.293 ± 0.052	4.014
K417N/E484K/N501Y	250.634 ± 26.257	4042.484	323.017 ± 5.775	4424.890
K417T/E484K/N501Y	59.937 ± 2.497	966.726	68.525 ± 8.03	938.699
L452R/T478K/P681R	0.146 ± 0.003	2.355	0.113 ± 0.021	1.548

^*a*^The experiments were performed in triplicate and repeated three times, and data are expressed as the means ± SD.

### Identification of K417N/T mutation as a key determinant of LCB1 resistance

In order to identify the mutations responsible for the VOC resistance, we further generated a panel of S protein mutants carrying the key mutations by VOCs. The corresponding pseudoviruses were generated and their infectivity was characterized on both 293T/ACE2 and Huh-7 cells by a single-cycle infection assay (Supplementary Figure 2). In the inhibition of pseudoviruses with single mutations, we found that LCB1 inhibited the infection of K417N mutant with IC_50_ of 2.774 nM on 293T/ACE2 and 4.898 nM on Huh-7 cells, which indicated 45-fold or 67-fold resistance changes over D614G reference (Figures 2C,D). While N501Y mutant showed mild resistance (∼4-fold), the single mutation of E484K or P681H and the mutant with Δ69-70 deletion did not confer resistance, which were also supported by the IC_50_ fold-changes of three combination mutations (Δ69-70/N501Y, N501Y/P681H, and Δ69-70/N501Y/P681H). Significantly, the E484K/N501Y mutant was more resistant than the N501Y mutant and its combination with K417N or K417T sharply enhanced the resistance phenotype, as evidenced by the fold-changes of two triple mutations (K417N/E484K/N501Y and K417T/E484K/N501Y). In contrast, the triple mutant L452R/T478K/P681R had no obvious resistance to the LCB1 inhibition. These results demonstrated that K417N or K417T mutation in RBD plays a key role in the resistance profiles of SARS-CoV-2 VOCs to LCB1 inhibitor. In line with this conclusion, the Beta, Gamma, and Omicron variants contain K417N or K417T, whereas the Alpha, Delta, and Lambda variants lack this mutation.

### Identification of naturally occurring receptor binding domain mutations that confer the LCB1 resistance

Considering the ongoing mutations of SARS-CoV-2 variants, we are interested in characterizing the effects of naturally occurring mutations on the inhibitory activity of LCB1. Thus, a total of 85 substitutions, which naturally occurred in the spike RBD sequence with relatively higher frequencies, were selected and the corresponding S protein mutants were generated. First, we analyzed the functionality of the S protein mutants to mediate pseudovirus infections on both 293T/ACE2 and Huh-7 cells. As shown in Supplementary Figure 3, many of the mutants displayed significantly decreased infectivity, whereas several mutations resulted in enhanced infections (L452M, I468T, Y505W, and Y508H). Herein, we determined the susceptibility of 81 pseudoviruses to the LCB1 inhibition on Huh-7 cells by the single-cycle infection assay. As indicated by the IC_50_ values in Table 2, there were three mutants (E406Q, K417T, and L455F) displaying high-levels of resistance with the fold-changes of IC_50_ at ∼31, ∼25, and ∼121, respectively, whereas the Y505W mutant conferred a mild resistance with a ∼6-fold increased IC_50_ value. In this experiment, we also verified that L452R and T478K mutations, which appeared in the L452R/T478K/P681R mutant above, had no resistance to LCB1, whereas some mutants (P463S, V483F, A520V, and P521R) behaved with certain degrees of increased susceptibility.

**TABLE 2:** Characterization of naturally occuring RBD mutations that mediate LCB1 resistance.

Pseudovirus	IC_50_ ± SD (nM)	Fold change	Pseudovirus	IC_50_ ± SD (nM)	Fold change
D614G	0.081 ± 0.025	1.000	G446V	0.039 ± 0.015	0.485
P330S	0.100 ± 0.014	1.250	L452M	0.084 ± 0.024	1.695
P337S	0.058 ± 0.019	0.714	L452R	0.136 ± 0.014	1.052
F338L	0.081 ± 0.026	1.013	Y453F	0.114 ± 0.026	1.424
G339D	0.035 ± 0.021	0.350	**L455F**	**9.802 ± 0.686**	**121.012**
V341I	0.116 ± 0.026	1.448	S459Y	0.039 ± 0.003	0.489
A344S	0.077 ± 0.021	0.964	P463S	0.018 ± 0.015	0.219
A348S	0.042 ± 0.006	1.752	I468T	0.068 ± 0.019	0.843
A352S	0.114 ± 0.012	1.419	T470A	0.044 ± 0.006	0.552
N354D	0.083 ± 0.021	1.032	E471Q	0.042 ± 0.007	0.519
N354K	0.066 ± 0.039	0.828	I472V	0.063 ± 0.015	0.784
N354S	0.077 ± 0.012	0.956	A475V	0.061 ± 0.017	0.764
S359N	0.077 ± 0.019	0.959	G476S	0.092 ± 0.014	1.152
V367F	0.088 ± 0.028	1.103	S477I	0.060 ± 0.015	0.748
V367L	0.098 ± 0.008	1.590	S477N	0.092 ± 0.023	1.155
N370S	0.077 ± 0.012	0.956	S477R	0.049 ± 0.004	0.609
A372T	0.082 ± 0.038	1.022	T478I	0.078 ± 0.008	0.974
S373L	0.082 ± 0.036	1.021	T478K	0.078 ± 0.009	0.975
F377L	0.050 ± 0.003	0.630	P479S	0.070 ± 0.017	0.880
K378N	0.086 ± 0.009	1.070	G482S	0.057 ± 0.007	0.716
V382L	0.054 ± 0.026	0.675	V483A	0.066 ± 0.005	0.823
P384A	0.084 ± 0.015	1.046	V483F	0.016 ± 0.007	0.199
P384L	0.088 ± 0.014	1.105	V483I	0.084 ± 0.005	1.044
P384S	0.049 ± 0.024	0.617	G485S	0.083 ± 0.013	1.043
T385A	0.096 ± 0.011	1.231	G485R	0.045 ± 0.014	0.560
T385I	0.092 ± 0.003	1.145	F486L	0.096 ± 0.026	1.200
T393P	0.082 ± 0.011	1.157	F490S	0.041 ± 0.009	0.512
V395I	0.091 ± 0.018	1.134	F490L	0.049 ± 0.010	0.617
I402V	0.088 ± 0.052	1.097	Q493L	0.046 ± 0.013	0.580
**E406Q**	**2.489 ± 0.299**	**31.125**	S494P	0.083 ± 0.019	1.043
R408I	0.096 ± 0.033	1.200	N501T	0.105 ± 0.009	1.306
Q409E	0.077 ± 0.020	0.956	V503F	0.064 ± 0.005	0.798
A411S	0.042 ± 0.006	0.527	**Y505W**	**0.479 ± 0.030**	**5.914**
Q414R	0.029 ± 0.002	0.361	Y508H	0.101 ± 0.011	1.263
**K417T**	**1.988 ± 0.558**	**24.543**	E516Q	0.070 ± 0.005	0.879
D427Y	0.080 ± 0.040	0.998	A520S	0.076 ± 0.006	0.954
A435S	0.045 ± 0.011	0.565	A520V	0.013 ± 0.006	0.167
N439K	0.033 ± 0.008	0.416	P521R	0.023 ± 0.003	0.289
N440K	0.069 ± 0.012	0.860	P521S	0.053 ± 0.017	0.657
K444N	0.042 ± 0.013	0.528	A522S	0.039 ± 0.004	0.488
K444R	0.046 ± 0.013	0.570	A522V	0.032 ± 0.016	0.404

^*a*^The experiments were performed in triplicate and repeated three times, and data are expressed as the means ± SD. The resistance mutations are highlighted in bold.

### The resistance mutations greatly impair the binding affinity of LCB1

**FIGURE 3:**
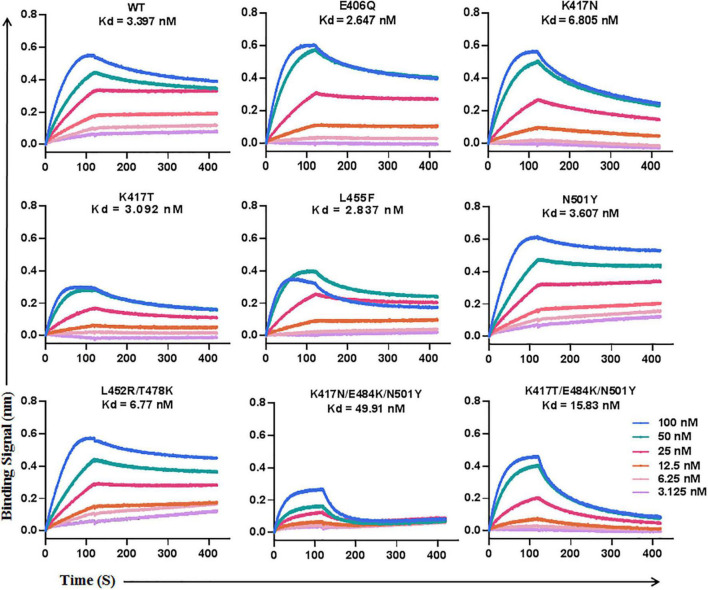
The binding affinity of LCB1 with RBD proteins determined by biolayer interferometry. A recombinant His-tagged RBD protein with wild-type sequence or mutations was loaded onto an NTA biosensors and equilibrated before the baseline was set to zero at *t* = 0. The binding kinetics was guided by associating in different concentrations of LCB1 for 120 s and disassociating for 300 s. The equilibrium dissociation constant (Kd) was calculated.

To explore the mechanism underlying the LCB1 resistance, we applied biolayer interferometry (BLI) to analyze the binding affinity of LCB1 to the RBD proteins with or without resistance mutations (Figure 3). Comparing to the equilibrium dissociation constant (Kd) of wild-type RBD (WT-RBD) at 3.397 nM, only the RBD proteins carrying a single K417N or double L452R/T478K mutations exhibited slightly reduced binding affinities with Kd values of 6.805 and 6.77 nM, respectively, none of other single mutations (E406Q, K417T, L455F, and N501Y) significantly impaired the LCB1 binding. Notably, the RBD proteins with the combination mutations of K417N/E484K/N501Y or K417T/E484K/N501 had dramatically decreased binding capacities as indicated by their Kd values at 49.11 and 15.83 nM, respectively.

**FIGURE 4:**
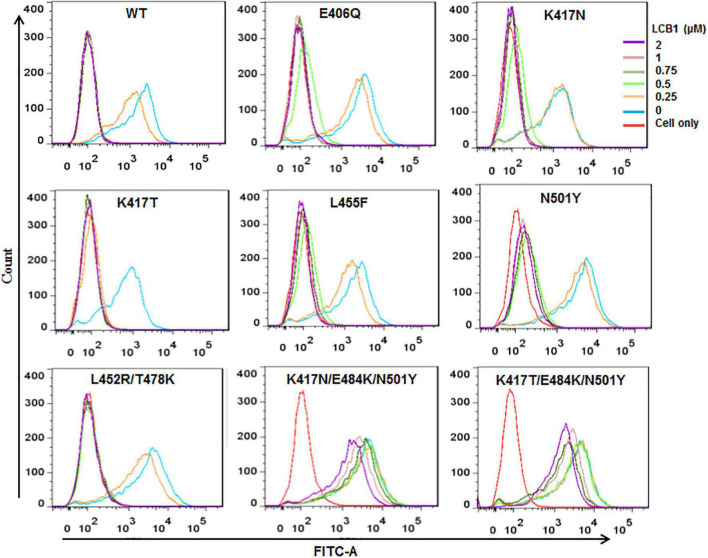
The blocking activity of LCB1 on the binding of RBD protein with the cell receptor ACE2. An His-tagged RBD protein was incubated with serially diluted LCB1 and then added to 293T/ACE2 cells for incubation. The bound RBD was detected by an Alexa Fluor 488-labeled rabbit anti-His tag antibody with FACS analysis.

We next determined the blocking activity of LCB1 on the binding of RBD protein with ACE2 expressed on 293T/ACE2 cells by flow cytometry. Consistent with its binding affinity above, LCB1 could effectively blocked the binding of RBD proteins with single or double mutations, but it exhibited a dramatically decreased blocking activity on the RBD with K417N/E484K/N501Y or K417T/E484K/N501Y (Figure 4).

## Discussion

In this study, we focused on characterizing LCB1 inhibitor for its antiviral activities against divergent SARS-CoV-2 variants and achieved significant findings. First, we successfully synthesized and purified a 56-mer LCB1 peptide, which exhibited high α-helicity, thermostability and antiviral activity. Second, we found that while the Alpha, Delta, and Lambda variants only caused mild resistance to LCB1, the Beta, Gamma, and Omicron variants were highly resistant to the LCB1 inhibition, giving the resistance fold-changes greater than 15,000 or 3,000. Third, with a panel of SARS-CoV-2 mutants carrying the single, double or triple mutations contained in VOCs, K417N, and N501Y in the spike RBD were verified to be critical determinants for the observed resistance phenotype. Fourth, we further generated a large panel of 85 naturally occurring RBD point-mutations, which identified E406Q, K417T and L455F conferring high-levels of resistance, whereas Y505W was responsible for a mild resistance. Moreover, the results by BLI and flow cytometry demonstrated that the resistance mutations could greatly impair the RBD-binding affinity of LCB1 and thus attenuated its blocking function on the RBD-ACE2 interaction. In conclusion, our studies highlight the resistance profiles of divergent SARS-CoV-2 VOCs and natural RBD mutants as well as the underlying mechanisms, which would facilitate the development of LCB1-based antiviral drugs.

Since its first report, LCB1 inhibitor has specially attracted our attention due to its computer-aided design strategy as a miniprotein and high inhibitory potency on SARS-CoV-2 infection by targeting the spike RBD to block virus entrance (Cao et al., 2020). We are also encouraged by the preventive and therapeutic efficacies of modified LCB1 proteins in animal infection models (Case et al., 2021). Unfortunately, SARS-CoV-2 spread with persistent mutations, resulting in the emergence of divergent VOCs and VOIs that caused new waves of worldwide epidemic. Significantly, many of evolved mutations in RBD can shape the binding conformation and interacting affinity of S protein with the cell receptor ACE2 and thus affect virus’s biological properties, e.g., transmission ability and disease severity. The mutations have also affected the performances of vaccines, therapeutic drugs, and diagnostic tools significantly (Garcia-Beltran et al., 2021; Hoffmann et al., 2021; Kuzmina et al., 2021; Fan et al., 2022; Guo et al., 2022; Sun et al., 2022; Zhu et al., 2022). Omicron has emerged as the fifth VOC after Alpha, Beta, Gamma, and Delta, and it has evolved into distinct lineages: BA.1 (B.1.1.529) was responsible for the initial surge but almost replaced by BA.2 in April 2022; while BA.3 remains at low frequency, BA.4 and BA.5 have currently replaced BA.2 and are becoming prevalent globally (Qu et al., 2022; Tegally et al., 2022). In protein level, BA.4 and BA.5 bear identical S proteins, being most comparable to BA.2 but have additional mutations (Δ67-70, L452R, F486V) and wild type amino acid at position Q493, which critically determine their increased fitness and immune evasion than the earlier BA.1 and BA.2 lineages (Cao et al., 2022). In this study, we identified that single K417N or K417T mutation is a key determinant to the resistance, which occurred in Beta, Gamma and Omicron (VOCs highly resistant to LCB1) but not in Alpha, Delta, and Lambda variants (VOCs or VOI mildly resistant to LCB1). Except for Delta, other four VOCs have an N501Y mutation, which is responsible for a low resistance level when being presented in the Alpha variant alone but can markedly boost the resistance levels of three highly resistant VOCs as a combined mutation with K417N/T. The Delta variant evolved with two RBD mutations (T478K and L452R); while T478K was not associated with the LCB1 resistance, L452R did rendered the virus slightly resistant, as evidenced by ∼2-fold IC_50_ changes (Table 2). L452R also appeared in Delta, Omicron, and many other variants, such as Kappa (B.1.617.1), Epsilon (B.1.429), and B.1.617.3. Our studies with the large panel of naturally occurring RBD mutants also found E406Q, L455F, and Y505W being resistant to LCB1, which are not present in the current VOCs. Given that all the characterized mutations in LCB1-resistant VOCs, including K417N, K417T, T478K, L452R, and N501Y, naturally emerged during the evolution process, whether E406Q, L455F, and Y505W mutations will appear in future VOCs to cause LCB1 resistance is an intriguing question.

In order to overcome the resistance problem by LCB1, the same group of authors continued their efforts to design multivalent proteins by using a cell-free expression workflow, which combines an *in vitro* DNA assembly step followed by polymerase chain reaction (PCR) to generate linear expression templates that are used to drive cell-free protein synthesis and enable rapid prototyping of new minibinder designs (Hunt et al., 2022). Promisingly, a number of constructs containing LCB1 or other miniproteins displayed dramatically increased binding and inhibitory activities against divergent VOCs and related mutant viruses, and of them a homo-trimeric version of 75-residue ACE2 mimic AHB2 (TRI2-2), which was designed to geometrically match the trimeric spike architecture, conferred prophylactic and therapeutic protection against SARS-CoV-2 challenge when administered intranasally in mice, providing evidence to support LCB1-based drug development with high and broad-spectrum antiviral activities (Hunt et al., 2022). In addition to developing viral entry inhibitors that target membrane fusion step (Zhu et al., 2019, 2020, 2021, 2022; Yu et al., 2021a,b; Xue et al., 2022), our research team is also working to rationally construct LCB1-based inhibitors that have resistance to viral escape and antigenic drift, and several bispecific fusion proteins have been validated with highly potent activity against diverse SARS-CoV-2 VOCs and mutants (unpublished data). Very recently, it was also reported that the length of LCB1 could be reduced to 35 amino acids without interfering its inhibitory capacity, providing a flexible template for LCB1-based design strategies (Weissenborn et al., 2022).

## Data availability statement

The original contributions presented in this study are included in the article/Supplementary material, further inquiries can be directed to the corresponding author.

## Author contributions

TW, YZ, NL, YH, and HC performed the experiments. TW, YZ, and YXH analyzed the data. YH supervised the study and wrote the manuscript with TW and YZ. All authors read and approved the submitted version.

## Footnotes

1
https://covid19.who.int

## Supplementary material

Supplementary Figure 1 Synthesis and characterization of LCB1 peptide. **(A)** LCB1 was chemically synthesized on rink amide 4-methylbenzhydrylamine (MBHA) resin using a standard solid-phase 9-flurorenylmethoxycarbonyl (FMOC) and its purity was determined by reverse-phase HPLC. **(B)** The peptide was characterized by mass spectrometry, indicating a molecular weight of 6,852.5Da.

Supplementary Figure 2 Infectivity of divergent SARS-CoV-2 variants. The infectivity of SARS-CoV-2 pseudoviruses with single or multiple mutations on 293T/ACE **(A)** or Huh-7 **(B)** cells was determined by a single-cycle infection assay. D614G mutant was treated as a reference, thus its luciferase activity (RLU) was standard as 100% and the relative infectivity of various mutants was calculated accordingly. The experiments were repeated at least three times and columns are expressed as the means ± SD. Statistical analysis was conducted to compare the differences between the D614G reference and diverse mutants.

Supplementary Figure 3 Infectivity of naturally occurring SARS-CoV-2 mutants. The infectivity of 85 SARS-CoV-2 pseudoviruses carrying natural RBD point-mutations was determined on 293T/ACE **(A)** or Huh-7 **(B)** cells by a single-cycle infection assay. Similarly, D614G mutant was treated as a reference, thus its luciferase activity (RLU) was standard as 100% and the relative infectivity of various mutants was calculated accordingly. The experiments were repeated at least three times and columns are expressed as the means ± SD. Statistical analysis was conducted to compare the differences between the D614G reference and diverse mutants.
