# Supplementary figures and images for: Resistance profile and mechanism of severe acute respiratory syndrome coronavirus-2 variants to LCB1 inhibitor targeting the spike receptor-binding motif
Source: Front Microbiol. 2022 Oct 11;13:1022006. doi: 10.3389/fmicb.2022.1022006 (PMC9593036; doi:10.3389/fmicb.2022.1022006)

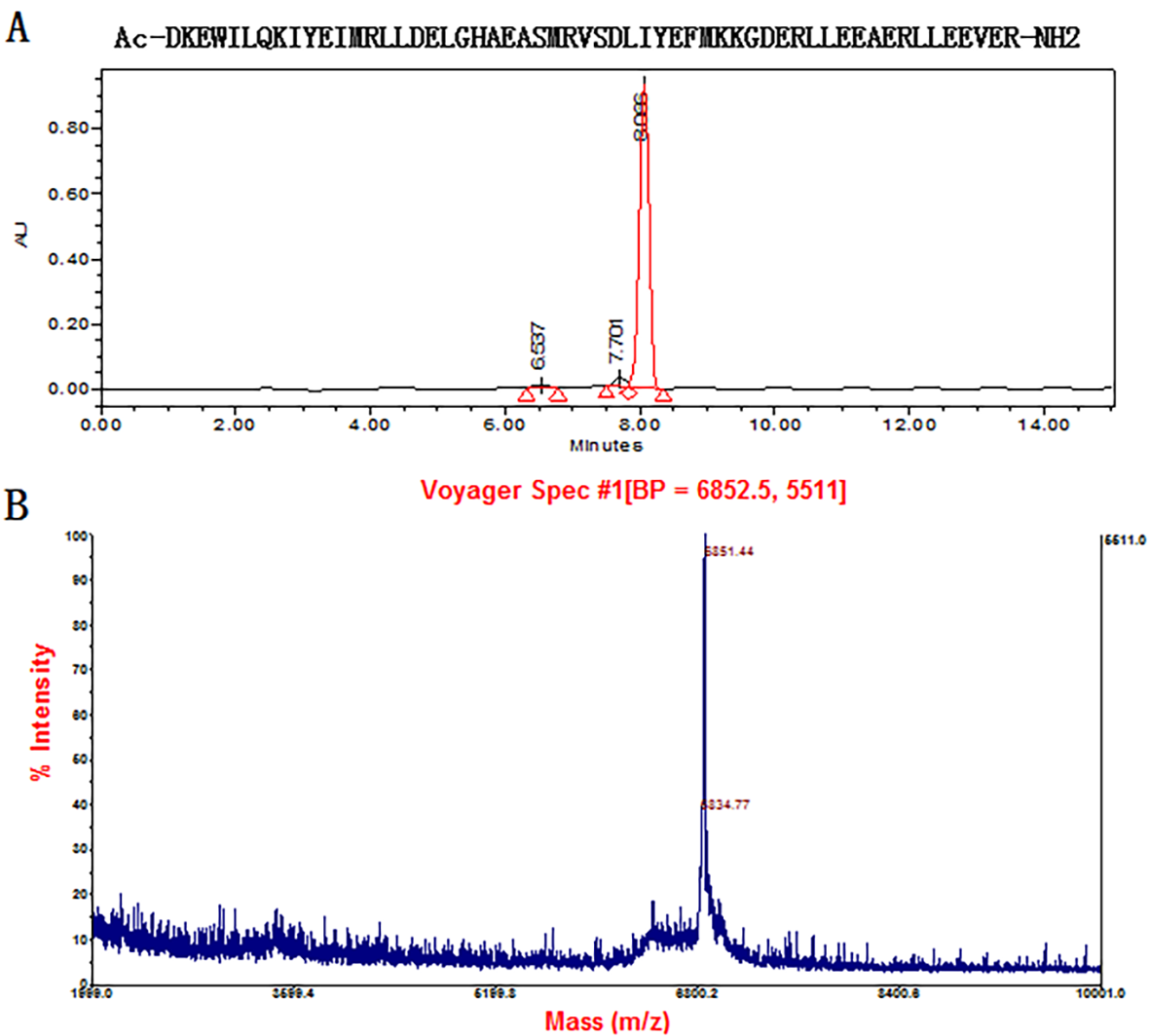

Supplement: Supplementary Figure 1 — Synthesis and characterization of LCB1 peptide. (A) LCB1 was chemically synthesized on rink amide 4-methylbenzhydrylamine (MBHA) resin using a standard solid-phase 9-flurorenylmethoxycarbonyl (FMOC) and its purity was determined by reverse-phase HPLC. (B) The peptide was characterized by mass spectrometry, indicating a molecular weight of 6,852.5Da. [file Image_1.TIF]

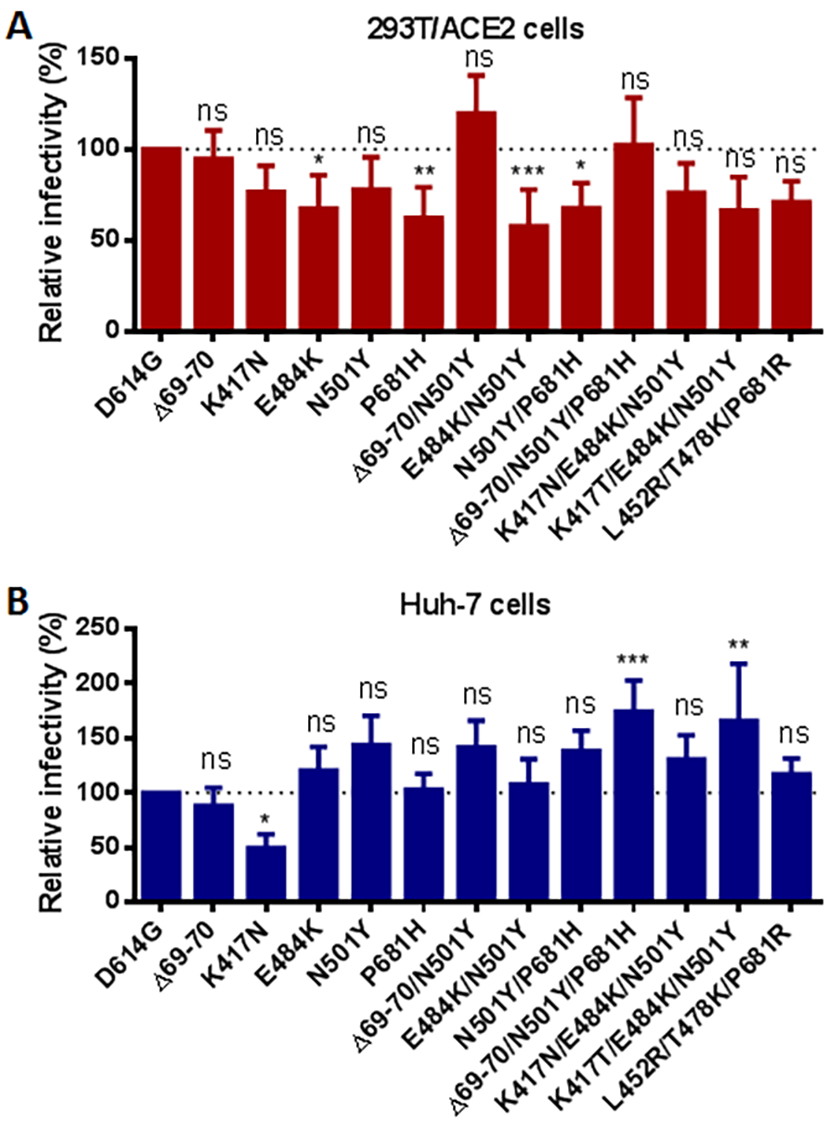

Supplement: Supplementary Figure 2 — Infectivity of divergent SARS-CoV-2 variants. The infectivity of SARS-CoV-2 pseudoviruses with single or multiple mutations on 293T/ACE (A) or Huh-7 (B) cells was determined by a single-cycle infection assay. D614G mutant was treated as a reference, thus its luciferase activity (RLU) was standard as 100% and the relative infectivity of various mutants was calculated accordingly. The experiments were repeated at least three times and columns are expressed as the means ± SD. Statistical analysis was conducted to compare the differences between the D614G reference and diverse mutants. [file Image_2.TIF]

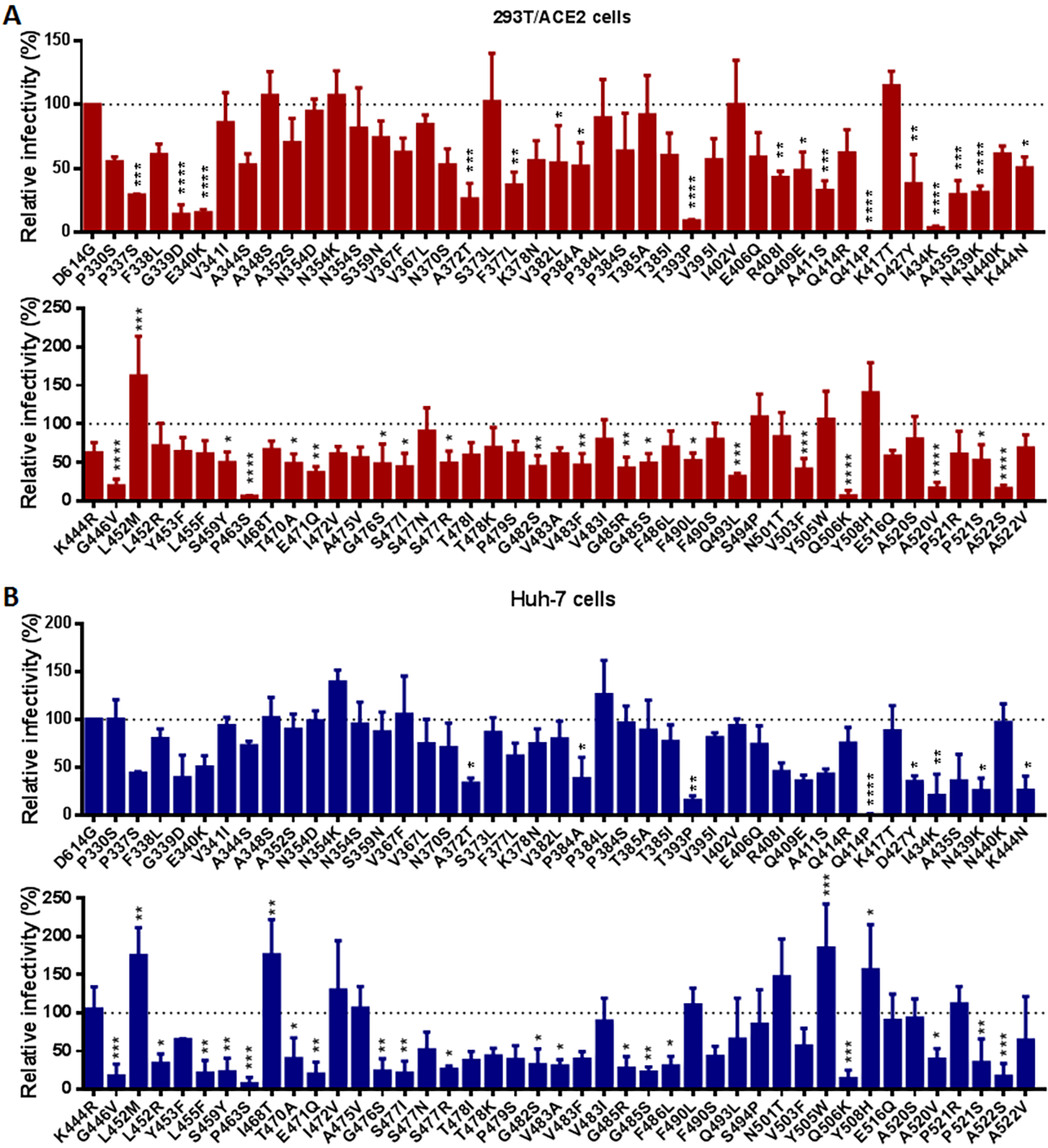

Supplement: Supplementary Figure 3 — Infectivity of naturally occurring SARS-CoV-2 mutants. The infectivity of 85 SARS-CoV-2 pseudoviruses carrying natural RBD point-mutations was determined on 293T/ACE (A) or Huh-7 (B) cells by a single-cycle infection assay. Similarly, D614G mutant was treated as a reference, thus its luciferase activity (RLU) was standard as 100% and the relative infectivity of various mutants was calculated accordingly. The experiments were repeated at least three times and columns are expressed as the means ± SD. Statistical analysis was conducted to compare the differences between the D614G reference and diverse mutants. [file Image_3.TIF]
